# Football and Dementia: Understanding the Link

**DOI:** 10.3389/fpsyt.2022.849876

**Published:** 2022-05-27

**Authors:** James Neal, Paul B. Hutchings, Ceri Phelps, Donald Williams

**Affiliations:** ^1^Institute of Life Sciences, Swansea University Medical School, Swansea, United Kingdom; ^2^Centre for Psychology and Counselling, Institute of Education and Humanities, University of Wales Trinity Saint David, Swansea, United Kingdom; ^3^Swansea University Medical School, Swansea, United Kingdom

**Keywords:** football, soccer, dementia, etiology, brain size, brain fragility

## Abstract

Football, also known as soccer or association football, is popular but has a potential link with dementia developing in retired players. The FA and soccer regulators in the USA have imposed guidelines limiting players exposure to heading, despite controversy whether this dementia is caused by heading the ball, a form of mild repetitive head injury (RHI), over many years. Substantial data exist showing that many ex-North American Football players develop a specific neurodegenerative disease: chronic traumatic encephalopathy (CTE), the neuropathological disorder of boxers. In the United Kingdom evidence for the neuropathological basis of footballers' dementia has been slow to emerge. A 2017 study revealed that in six ex-soccer players four had CTE with Alzheimer's disease (AD) and two had AD. A 2019 study showed that ex-footballers were 3.5 times more likely to die from dementia or other neuro-degenerative diseases than matched controls. We argue that in childhood and adolescence the brain is vulnerable to heading, predicated on its disproportionate size and developmental immaturity. RHI in young individuals is associated with early neuroinflammation, a potential trigger for promoting neurodegeneration in later life. Evidence is available to support the guidelines limiting heading for players of all ages, while professional and non-players should be included in prospective studies to investigate the link between soccer and dementia.

## Introduction

Football, also known as soccer or Association football, is the most popular game in the world, played by ~250 million people in >200 countries; 22 million of these players are adolescents or younger. Globally ~47 m people were living with dementia and it is regarded as the greatest challenge for health and social care in the twenty-first century (1). The controversial links between playing soccer and subsequent dementia have been increasingly debated, after several studies have identified dementia in retired professional soccer players (2, 3). However, this effect has not been consistently replicated, especially in younger, non-professional players with less exposure to heading (4, 5). Despite the controversy the data underpinning this association was considered sufficiently persuasive by the Football Association (FA) authorities in the USA and UK to accept that a problem exists. This led to the introduction of guidelines limiting the frequency of heading a football during a week children and adult players are permitted to perform (6–9).

Young NFL players, boxers and soccer players are exposed to sub-concussive impacts or repeated head injury (RHI) at a critical time for brain development (10–12) potentially increasing the risk of long term cognitive injury. Although the link between heading a football (RHI) and dementia in soccer players is not proven, we discuss the evidence supporting the decision to limit heading in children and adolescents because of its short- and long-term effects on the brain.

## Background

The link between heading a football and subsequent dementia has been controversial for some 20 years. In 2002 a Coroner ruled that Jeff Astle, a powerful header, had died from an industrial disease: dementia caused by heading the ball over many years. There was no published material about a link between heading and subsequent dementia, although there were reports of “footballer's migraine” resulting from heading a traditional leather football (13). Since this landmark verdict a litany of high-profile players have been reported to be suffering from or having died from dementia. Four of the 1966 England World Cup winning team have died recently from dementia.

## Soccer, Boxing and CTE

The association between RHI and development of neurological and cognitive symptoms has a precedent in retired boxers suffering from a syndrome known historically as “Punch drunk” or “dementia pugilistica,” and now as Chronic Traumatic Encephalopathy (CTE). The clinical presentation of CTE in retired boxers involved memory loss and motor impairment with some degree of aggression and cognitive decline (13–17). A description of the distinctive neuropathology of CTE in boxers was provided by Corsellis et al. (17) and this has been gradually refined such that CTE is now regarded as a distinct neurodegenerative disease and an example of a primary tauopathy (18). CTE still can only be diagnosed at autopsy; several retrospective studies involving retired contact sports and professional North American football (NFL) players have linked the development and severity of CTE with prolonged exposure to RHI and this provides the best model available for investigating a link between delayed dementia and RHI in soccer (19–21).

The parallel between heading a football, involving RHI, and boxing, both with repeated exposure to sub-concussive impacts (RHI), is compelling. Boxing and sparring sessions are analogous to heading practice in soccer with cumulative RHI over decades (17). A comparison between the forces generated by impacts to the head during boxing and sparring found the peak angular and peak linear acceleration values were similar for both and within the range generated by a soccer ball traveling at 12–54 m/s impacting on the head (22–24). Peak acceleration values at the surface of the head after heading in adolescent soccer players was found to 160–180% greater than non-injurious impacts during hockey or American football, where the players wear helmets. Boxers and soccer players experience RHI predominantly distributed over the front of the head (22–24). These data should be considered with regards to their cumulative effects upon the developing brain in young soccer players and boxers (10–12).

## American Football: Links With CTE and Dementia

American NFL footballers (18–21), soccer players (19, 25–28), and boxers (17) with CTE share the same underlying neuropathological changes, namely septal fenestration and a characteristic distribution of the neuronal protein hyperphosphorylated tau, mixed 3 and 4 repeat (p-tau) isoforms (3R/4R) around small diameter cerebral blood vessels in the depths of the cortical sulci, within neurons, p tau + or—astrocytes and as neurofibrillary tangles (NFT); β-amyloid plaques are not a feature of CTE (18–20). The severity of p-tau neuropathology is progressive throughout the brain (stages I–IV) and corresponds to increasing cognitive impairment and aggressive behavior (18–20, 29). Axonal injury and loss are associated with accumulation of p-tau and is present in all stages of CTE progressing to involve white matter, medial temporal lobe, thalamus and brainstem (19, 20).

In one retrospective series of 85 individuals with mild repetitive traumatic brain injury, 65 were diagnosed neuropathologically with CTE; of these 37% were found to have marked accumulation of Trans active response -DNA binding protein-43 (TDP-43), α-synuclein and β-amyloid in late stage CTE; these proteins could contribute to the clinical symptoms of CTE (20). The authors speculated RHI associated with axonal injury could trigger different molecular pathways resulting in the accumulation of misfolded p-tau and other brain proteins, TDP-43, α-synuclein and β-amyloid explaining the high proportion of CTE cases with concomitant neurodegenerative disease (Motor Neuron Disease, Lewy Body Disease, Alzheimer's Disease) (20, 30). A significant correlation was noted between years after retirement age at death and CTE stage, a measure of increasing pathological severity with survival (18, 20). The number of concussions, educational attainment and use of anabolic steroids did not correlate with CTE stage (18, 20), whereas a larger retrospective study of 202 deceased NFL players correlated changes of mood, cognition, and dementia with severe neuropathology (29).

From a cohort of >1,700 male non-tauopathy disease formalin fixed cases, 66 were identified retrospectively having a history of contact sport; of these only eight were professional or semi-professional sports players (31). Over 30% (21) of the 66 cases met NINDS neuropathological criteria for CTE, 45 did not have CTE (18), two thirds had mild/moderate stage neuropathology (I–II) and one third were stages III–IV. A significant number of cases from the same cohort with a history of a single TBI (fall, assaults) but not contact sport, did not have CTE pathology. The authors concluded that exposure to contact sports (RHI) was a high risk for CTE neurodegenerative pathology and validated the NINDS criteria for CTE neuropathology (18). These data were confirmed by showing the odds of a player getting CTE doubled every 2.6 years of playing NFL and was accompanied by increased severity, representing a measure of the cumulative exposure to RHI, i.e., a dose-response effect (32).

## A Note of Caution Linking RHI With CTE

To accurately correlate neuropathology with the various traumatic, medical, alcohol and drug exposures during an individual's lifetime is challenging (20, 33, 34). Retrospective studies involving professional NFL players introduces ascertainment bias as families with deceased exhibiting cognitive symptoms are more likely to donate tissue compared with those related to a normal cognitive individual (20, 33). It is essential individuals exposed to RHI with no behavioral or cognitive symptoms are included in future studies (20). Obtaining retrospective data from medical records or next of kin, without a standardized collection protocol, adds to the difficulty of identifying a core group of psychiatric symptoms that define CTE, especially in individuals without a history of RHI or concurrent AD (33). Furthermore, diagnostic symptoms for CTE are extensive and are not required to have a delayed onset or progressive profile, an unusual clinical presentation for a neurodegenerative disease (29).

## Interpretation of Criteria for CTE Neuropathology

Recent critical reviews have criticized the accuracy of the correlation between the clinical and neuropathological features of CTE (33, 35, 36). One study found CTE in individuals with no history of RHI (36) and a larger study of retired professional boxers and athletes dying in their 80s (20) retrospectively diagnosed early CTE (stage I–II) which is inconsistent with a typical progressive neurodegenerative disease (29, 30). Significant number of retired players with CTE stages (I–II) were associated with accumulation of TDP−43, β-amyloid, and cerebrovascular disease. In these cases, early stage CTE was regarded as an incidental finding or comorbidity, rather than the primary cause of clinical dementia (28). On this basis it is not clear whether select combinations of CTE and concomitant neuropathology influence clinical presentation and neurological signs (33).

One study of 268 cases of neurodegenerative disease and control cases reported 12% had neuropathology of CTE, mostly stage I–II (37). A small longitudinal study identified individuals with pathological changes of CTE but without a history of TBI or RHI (36). Subsequent analysis of the above series (36, 37) found the pathological changes of CTE either did not meet the NINDS criteria or were confused with Age Related Tau Astrogliopathy, a common pathological finding in elderly brains (18, 36–39).

## Recent Research Linking Soccer With CTE and Neurodegenerative Disease

In a prospective study of >7,000 professional soccer and basketball players and cyclists, only the footballers were found to be significantly at risk of developing ALS, possibly due to repetitive heading or prolonged physical exercise creating episodes of microtrauma (40). A young soccer player heading a football since age 3 died aged 29 years with ALS (TDP 43+) and early neuropathological CTE changes (21).

Isolated neuropathological reports of retired soccer players with dementia (20, 25, 26) were reported before Ling et al. (27) examined a series of fourteen retired players (24); all had long standing (average 26 years) exposure to heading commencing in childhood. Impacts associated with concussion and head-head collisions were rare. unlike NFL players. Neuropathological examination of six brains found that all had AD, four with associated CTE; there were similarities with NFL players and boxers, a high incidence of septal fenestration (13, 17, 19, 20), TDP-43 and β-amyloid accumulation. Two earlier cases of retired soccer players, both exposed to frequent heading, and both had neuropathological evidence of CTE (25, 26). Two separate studies of retired soccer players (all <60 years of age) found cognitive decline; in one Magnetic Resonance Imaging (MRI) evidence for cortical thinning and the other abnormal electrophysiological activity, respectively, and interpreted this as evidence for an underlying neuro degenerative process (41, 42).

The concomitant finding of CTE and AD was considered to be related to prolonged exposure to heading although a causal link could not be confirmed (27). An epidemiological study of 7,676 former soccer players and matched controls (43) found players were 3.5 times more likely to die from a dementia or neurodegenerative disease. The authors concluded “evidence supports an association between elite level participation in contact sports and increased risk of neurodegenerative disease, which, on balance of probabilities is a consequence of exposure to repeated head impacts” (43). This important study requires replication with consideration of additional risk factors for dementia (alcohol, drug use, cardiovascular disease) although the prevalence of neurogenerative disease was only 2% of the soccer players in the study (43).

Subsequent analysis found the risk of neurodegenerative disease was highest in defenders where heading is a prominent skill; the risk was also linked with career length (44). Goalkeepers were found to live longer than outfield players and to have a lower risk of developing neurodegenerative disease, both effects being attributed to a lower cumulative exposure of goalkeepers to RHI (45). These dose-response findings in soccer are in accord with the correlation between career length and development of CTE in NFL players (32).

## Is the Vulnerability of the Brain to the Effects of RHI due to Evolution and Prolonged Development?

The modern human brain evolved from our last common ancestor 5–8 million years ago; the human brain is three times bigger than expected for an ape of the same body weight and on average 60% heavier, controlled for body weight, compared to our closest living relative, the chimpanzee (46). This increase in brain weight/body weight ratio corresponds to the evolution of bipedalism and a hunter-gatherer existence reliant upon cognitive skills. These adaptations made the human brain particularly vulnerable to the effects of head trauma, especially rotational and linear movements as found in modern contact sports and exemplified by heading a football (22, 24, 46, 47).

Between late childhood and adolescence, interconnected cortical areas important for future complex social and intellectual behaviors, the so-called “social brain” undergo myelination, synaptic organization and cortical growth (11, 12, 48–50). Given the inherent fragility of the brain, exposure to RHI during critical periods of brain development has the potential to disrupt the protective effects of cognitive reserve (CR). This term describes the brain's capacity to compensate against the detrimental effects of aging and other risk factors associated with dementia through synaptic plasticity/remodeling and efficient networking between interconnected brain areas (51–53). RHI exposure during critical periods of brain development could reduce the protective effects of CR (measured by occupational attainment). Exposure to tackling in NFL football before 12 years of age was significantly correlated with early onset of cognitive symptoms and impaired neuropsychological testing, but not with severity of CTE pathology (54, 55). The authors propose early exposure to RHI reduced CR and protection against “late life neuropathology” (55). This conclusion has been criticized after a large (>3,550) study of NFL players found no correlation between early age at first tackle with subsequent cognitive performance or behavioral problems (56).

## Repetitive Heading Is Associated With Reversible Short-Term Changes in Brain Function

The deleterious effects of RIH have been demonstrated in two acute exposure studies; one found transient electrophysiological and cognitive anomalies after heading a ball only 20 times (57) whereas the levels of serum neurofilament, a marker for axonal injury, were elevated 24 h after heading, implying axonal injury is present even after minimal contact (58). Exposure to heading over a 12-month duration found impaired memory and attention (59) and accorded with results of MRI performed over a 1-year period with adult soccer players after heading which demonstrated white matter micro-structural changes and contemporaneous poor memory scores (60). These studies found cognitive and structural changes returned to normal once heading has been stopped and supports the interpretation that the brain has the capacity to mitigate the short-term consequences of RHI (3–5). Cerebral plasticity in adult non-human primates and rodent models is well-described after TBI (61, 62). A study using quantitative EEG methodology found disrupted cortical connections after a single TBI were reorganized in conjunction with improvement in cognitive performance after 9 months of neurorehabilitation, but it is not clear if this represented a sustained functional response (63).

## RHI in Young Individuals Is Associated With Early and Persistent Neuroinflammation

Survivors of single and repetitive TBI are known to have an increased incidence of AD (30, 64) with the mean age of onset of cognitive decline for both AD and non-AD dementias lower when compared to onset of cognitive decline in AD without a history of TBI (64). This later association was independent of any association with a specific concomitant neurodegenerative disease including CTE, raising the possibility that TBI in younger individuals initiates a global brain injury response, for example, multiple axonal disruption accompanied by activation of microglia (neuroinflammation) preceding significant accumulation of p-tau (18, 19, 65). This finding is consistent with a significant increase of tau + NFT and Aβ plaques in 30% of survivors under 60 years of age, at least 1 year after a single episode of moderate severe TBI; many were young adults in their third and fourth decade (65). A further four individuals (23–28 yrs) all exposed to RHI including soccer, had tau +NFT and tau threads located around blood vessels but no Aβ plaques; the distribution of p-tau resembled the earliest stage of CTE (19, 66).

After a single TBI survivors demonstrate persistent neuroinflammation in white matter tracts for over a year (67). Positron Emission Tomography (PET) scanning using the radioligand PK detected TSPO (mitochondrial translator protein) expressed by activated microglia up to 17 years after a single TBI. The density of PK+ microglia in several cortical areas (not restricted to the site of injury) correlated with the severity of cognitive impairment (68).

Elevated numbers of activated microglia (neuroinflammation) were found in the frontal cortex from a retrospective cohort of 66 cases of NFL players, 48 with CTE (44–66 years) (68). The density of microglia correlated with a p-tau accumulation, with the duration of career length (most commencing 12–14 years of age) providing a proxy measure of exposure to RHI and indirectly with the increased risk of developing dementia, a dose-response effect (69). PET scanning *young* individuals (average age 26 years, male and female) post-TBI found persistent microglial and tau accumulation were present 6 months post injury (70). Frequent, mild TBI, such as heading, rather than a single TBI provides an effective stimulus for chronic activation of microglia and p-tau accumulation, by preventing axonal recovery between episodes of trauma (69, 71). A younger group of NFL players with less exposure to RHI demonstrated increased microglia density, but no p-tau deposition. These data are suggestive of an early phase of persistent microglia activation, preceding p-tau accumulation and neurodegeneration (i.e., early CTE) in young players (68). Activated microglial also promote hyperphosphorylation of tau and promote its distribution within the brain (72). The functional role for p-tau beyond representing a diagnostic maker is not yet clear, whereas severity of ongoing inflammation shows a dose-response effect related to RHI exposure and risk of dementia and a potential therapeutic target (32, 69).

## The Development of Biomarkers for Early Detection of Neurodegenerative Disease (CTE)

MRI scanning to examine brain structure has a limited application for diagnosing CTE (73). Using PET, with Flortaucipir, a 3R/4R tau isoform tracer, was able to distinguish NFL players with cognitive and psychiatric symptoms from non-TBI controls but did not associate with players cognitive and psychometric scores (74).

A fluid-based biomarker with sufficient sensitivity and specificity to be of practical use for identifying CTE is essential, a combined CSF p-tau 231/ Aβ 1-42 assay has shown promise distinguishing CTE from AD but limited to *postmortem* samples (75). A combined assay to measure levels of tau phosphorylated at threonine 181 in plasma for use in conjunction with a plasma neurofilament light chain assay to discriminate between AD and non-AD neurodegenerative dementias, for example CTE, is under development (76, 77). A plasma biomarker measuring longitudinal levels of activated microglial related molecules, rather than accumulation of p-tau, has potential for monitoring the progressive damaging effects of RIH outside a specialized clinic (72, 73).

## Recent Epidemiological Studies to Investigate Cognitive Performance and Concussion in Contact Sports Players

Current studies analyzing an association between concussion in contact sports and cognitive performance have inherent limitations (78). Many demonstrate variability of inclusion criteria, i.e., professional and non-professional sports players, no agreed definition of concussion (79) and exclusion of other risk factors for dementia (age, sex, educational attainment, cardiovascular, diabetes) (78). The individual studies employ different cognitive screening tests to investigate neurocognitive performance [e.g., Montreal Cognitive Assessment (MoCA) (80), Telephone Interview for Cognitive Status (TICS-m) (81); neuropsychological testing required to test all facets of cognitive performance is rarely used (34, 82)]. However, the importance of formal neuropsychological testing is demonstrated by a recent study that confirmed cognitive impairment is related to RHI in retired professional soccer players (83). Several studies regarded career length or the number of boxing bouts as a proxy measure of concussion but this is more likely to reflect cumulative RHI over a defined period rather than separate episodes of concussion (78). Prospective studies such as the Diagnostic Imaging Genetics Network for the Objective Study and Evaluation of Chronic Traumatic Encephalopathy (DIAGNOSE CTE) involving former NFL players and includes individuals with no history of contact sports participation (34). Two current prospective studies, HEADING (84) and REIMPACT (85) (soccer in 14–16 year olds), address issues in soccer and should incorporate many of the above recommendations such as standardization of neuropsychological and clinical examinations together with collation of neuropathological and imaging data (34). Undoubtedly one of the major challenges faced in protecting players of contact sports is the identification and use of a suitable cognitive task measure. For example, whilst the MoCA has been found to be a useful tool in identifying impairment in those with a history of concussion (86) there remain questions regarding its ability to distinguish cognitive impairment at a fine level for all individuals (87). Therefore, it is important that cognitive test developers are part of the wider discussion relating to the appropriateness of testing contact sports players.

## Prevention

Helmets are not able to prevent the deleterious effects of acceleration deceleration responsible for axonal injury in concussion (88). An innovative approach mitigating the effects of RHI is thorough the reduction of intracranial compliance (slosh effect) using external compression of the internal jugular vein (IJV) to increase intracranial venous volume. The overall effect is to reduce the relative motion of intracranial contents; in animal studies, this reduced axonal injury during trauma by over 80% (88, 89), microglial activation by up to 60% and neuronal loss was reduced by over 50% (88). Soccer players who wore a cervical collar IJV device over one season, demonstrated some protective effects against RIH (90).

## Conclusion

We discuss evidence to support the FA and US soccer regulators limiting heading, especially in young players, despite the link between soccer and dementia is “not yet proven.” The association between contact sports, e.g., NFL players with CTE is strong, but not universally accepted for reasons we discuss. Current efforts to develop robust biomarkers and well-designed, prospective epidemiological studies involving contact sports players from an early age to assess the risk of cognitive decline and develop therapy are essential. In the USA, the DIAGNOSE CTE and Concussion Legacy Foundation (91) projects are currently investigating the association between contact sports players (NFL, rugby, hockey, soccer) and CTE (34). A prospective study in the UK (HEADING) and the multinational REPIMACT study will examine a potential link between soccer players and dementia (84, 85). The results of these studies will be highly influential guiding the future regulation of all types of contact sports.

## Data Availability Statement

The original contributions presented in the study are included in the article/supplementary material, further inquiries can be directed to the corresponding author/s.

## Author Contributions

After DW and JN had prepared the first draft, CP and PH became involved. From this point all authors shared the work of re-writing and preparing the article for publication. All authors contributed to the article and approved the submitted version.

## Conflict of Interest

The authors declare that the research was conducted in the absence of any commercial or financial relationships that could be construed as a potential conflict of interest.

## Publisher's Note

All claims expressed in this article are solely those of the authors and do not necessarily represent those of their affiliated organizations, or those of the publisher, the editors and the reviewers. Any product that may be evaluated in this article, or claim that may be made by its manufacturer, is not guaranteed or endorsed by the publisher.
